# Airway management and one‐lung ventilation strategies during hepatic histotripsy: a retrospective service evaluation from a single institution in Hong Kong

**DOI:** 10.1002/anr3.70092

**Published:** 2026-07-20

**Authors:** M. H. M. Chu, C. Y. T. Hui, H. M. K. Wong

**Affiliations:** ^1^ Department of Anaesthesia and Intensive Care The Chinese University of Hong Kong Hong Kong SAR China; ^2^ Department of Anaesthesiology CUHK Medical Centre Hong Kong SAR China

**Keywords:** double‐lumen tube, hepatic histotripsy, one‐lung ventilation

## Abstract

This retrospective single‐centre service evaluation describes double‐lumen tube one‐lung ventilation as a respiratory motion‐control strategy for hepatic histotripsy in 26 adults in Hong Kong. Histotripsy is a non‐invasive, non‐thermal focused ultrasound technique requiring precise targeting, challenged by respiratory‐induced liver motion. High‐frequency jet ventilation has been used to minimise motion but carries practical and physiological limitations. All procedures were performed under general anaesthesia with intended double‐lumen tube one‐lung ventilation. Successful double‐lumen tube placement without modification was achieved in 81% of cases; overall, 92% of patients received one‐lung ventilation using either double‐lumen tube or bronchial blockers. Transient intra‐operative desaturation occurred in 29% of one‐lung ventilation cases but was managed with standard ventilatory adjustments. Technical success was achieved in 92% of cases. No major airway or respiratory complications or unplanned critical care admissions occurred and all patients were discharged within 2 days postoperatively. These findings suggest that double‐lumen tube one‐lung ventilation is a feasible and safe alternative to high‐frequency jet ventilation for respiratory motion control during hepatic histotripsy, using widely available thoracic anaesthesia techniques. The retrospective design, small sample size and absence of comparator group highlight the need for future prospective trials to evaluate efficacy, safety and cost‐effectiveness relative to high‐frequency jet ventilation.

## Introduction

Histotripsy is a minimally invasive liver tumour therapy which uses focused ultrasound to mechanically fractionate tissue via controlled liquefaction without thermal injury [1, 2]. In October 2023, the US Food and Drug Administration granted marketing authorisation for the Edison histotripsy system (HistoSonics, Inc., Minneapolis, MN, USA) for non‐invasive treatment of unresectable primary and secondary liver tumours [3]. As primary liver cancer remains a major cause of cancer‐related mortality globally, effective minimally invasive treatments are needed [4, 5].

This non‐thermal, non‐ionising modality enables precise tumour destruction while preserving surrounding parenchyma and vascular structures. However, diaphragmatic excursions cause craniocaudal liver displacement, preventing consistent maintenance of the histotripsy focus on the target and thereby compromising treatment precision [6]. High‐frequency jet ventilation (HFJV) reduces hepatic motion but is associated with hypercapnia, haemodynamic instability, barotrauma risk and requires specialised equipment and expertise, restricting wider adoption [7, 8, 9, 10].

The published anaesthetic literature on hepatic histotripsy has focused on HFJV and the clinical trials of hepatic histotripsy (THERESA and #HOPE4LIVER) employed general anaesthesia without specifying the mode of ventilation [1, 2, 8]. There is a notable absence of published clinical data evaluating alternative ventilation strategies, specifically one‐lung ventilation (OLV), for this indication. One‐lung ventilation using double‐lumen tracheal tubes (DLT) or bronchial blockers is standard practice in thoracic anaesthesia for lung isolation and requires careful balancing of oxygenation, ventilation and lung‐protective settings [11]. Complete ipsilateral lung collapse reduces diaphragmatic motion while selective lung inflation can dynamically reposition lesions to optimise ultrasound targeting and avoid rib interference. However, the feasibility, safety and efficacy of DLT‐OLV for hepatic histotripsy have not been evaluated clinically.

To our knowledge, no clinical series has described a DLT‐OLV strategy for hepatic histotripsy in humans. This single‐centre retrospective service evaluation from Hong Kong describes the clinical use of DLT‐OLV as a motion‐control strategy for hepatic histotripsy and outlines the peri‐operative management pathway [12, 13, 14]. Our findings aim to inform other anaesthetists on alternative strategies of airway and ventilation management when providing care for patients undergoing hepatic histotripsy.

## Methods

This retrospective service evaluation included all consecutive adult patients undergoing hepatic histotripsy at CUHK Medical Centre (CUHKMC), Hong Kong, between April and October 2025. Patient characteristics and clinical information were retrieved from the CUHKMC Hospital Information System and, where applicable, the Hospital Authority Clinical Management System. Ethics approvals were obtained from the CUHKMC Ethics Committee (CREC Ref no: CREC‐202521; approved on 24 November 2025) and the Joint Chinese University of Hong Kong–New Territories East Cluster Clinical Research Ethics Committee (CREC Ref No: 2025.774; approved on 10/12/2025), with a waiver of informed consent for retrospective analysis of anonymised data.

We evaluated the complete initial institutional experience during the first 7 months of programme establishment. Patients undergoing additional surgical procedures during the same anaesthetic were not included in this analysis, in order to focus on cases where anaesthesia and ventilation were primarily tailored to histotripsy.

All histotripsy procedures were performed under general anaesthesia with the default airway plan of DLT‐OLV to reduce respiratory motion. During the period of this service evaluation, HFJV was not available at our institution; therefore, DLT‐OLV was adopted as a clinician‐led practice informed by multidisciplinary team discussion and established thoracic OLV strategies, rather than as part of a formal research protocol or institutional clinical pathway. Histotripsy had been introduced in another local hospital several months before the commencement of our service and our approach incorporated clinical experience shared from that early adopting centre. Patients were not specifically informed that this was a centre‐specific or novel technique in research terms, because it was implemented as part of individualised clinical care rather than as an experimental intervention. However, its use was embedded within standard peri‐operative decision‐making and institutional oversight for patient safety and quality assurance.

Anaesthesia was induced with intravenous propofol, an opioid (typically remifentanil or fentanyl) and a neuromuscular blocking agent according to anaesthetist preference, followed by tracheal intubation with a DLT under videolaryngoscopy. Anaesthesia was maintained either with total intravenous anaesthesia using propofol target‐controlled infusion (effect site concentration titrated to clinical signs or processed electroencephalogram, where available) combined with remifentanil infusion, or with sevoflurane (end‐tidal 1.5–2.5%) plus remifentanil infusion, according to anaesthetist preference. During OLV, tidal volume was generally set at 5–6 ml.kg^−1^ ideal body weight, with F_I_O_2_ titrated to maintain SpO_2_ ≥94%. Titration of tidal volume and ventilatory strategies was jointly discussed between the anaesthetist, interventional radiologist and surgeon, based on real‐time image quality and the patient's ventilatory status. Episodes of desaturation, when documented, were managed with increased F_I_O_2_, application of PEEP and recruitment manoeuvres to the ventilated lung and, when required, CPAP to the non‐ventilated lung, in line with standard thoracic anaesthesia practice.

The aim of this service evaluation was to describe our institutional practice and early experience with DLT‐OLV to facilitate liver histotripsy, including airway and ventilation strategies, technical aspects of histotripsy delivery and immediate peri‐operative course. We describe successful DLT placement without intra‐operative change in airway management, technical success of histotripsy, episodes of intra‐operative desaturation (SpO_2_ ≤94% during OLV), severe complications, ventilation parameters and procedural duration using descriptive statistics only. Continuous variables are reported as median (IQR [range]) or mean (SD) and categorical variables as counts (percentages). No inferential or hypothesis‐driven statistical testing was undertaken. Data were collated and tabulated using Microsoft Excel (Microsoft Corporation, Redmond, WA, USA).

## Results

In this retrospective service evaluation, 26 liver histotripsy procedures were identified and analysed (mean age 71 (62–80) years; 15 (58%) male). Hepatocellular carcinoma was present in 17 patients (65%) and metastatic liver disease in 9 (35%), with indexed lesions located across multiple hepatic segments (the largest lesion if there were more than one lesion).

Double‐lumen tube placement was attempted in all cases. Successful DLT insertion without airway plan modification was achieved in 21/26 patients (81%). In five patients (19%), DLT insertion failed: one was managed with a supraglottic airway and bilateral ventilation and four with single‐lumen tubes, of whom three received right‐sided bronchial blockers (OLV) and one remained on two‐lung ventilation (TLV). The two TLV cases received intentionally lower tidal volume (3 ml.kg^−1^) to minimise hepatic motion. Overall, 24/26 patients (92%) were managed with OLV.

Reasons for DLT insertion failure were anteriorly located larynxes with difficulties in DLT manipulation at vocal cord level (n = 4) and small airway calibre (n = 1). Technical ablation success was achieved in all five patients who did not receive DLT‐OLV and no additional airway or ventilation‐related complications occurred in this subgroup.

Among the 24 OLV cases, seven (29%) developed transient desaturation (SpO_2_ < 94%), all of whom were managed by using increased F_I_O_2_ and PEEP to the ventilated lung; in one case, low‐level CPAP was additionally applied to the non‐ventilated lung and no conversion to TLV was required. Median tidal volume during OLV was 5.5 ml.kg^−1^ (5.0–6.0 [4.1–7.3]), and median highest end‐tidal CO_2_ was 5.2 kPa (4.7–5.7 [4.0–7.0]); median procedure duration was 73 min (59–109 [19–243]) and median lesion size 2.4 cm (IQR 1.8–3.1 [1.0–5.7]). Technical success, defined as complete planned histotripsy coverage of the target lesion on immediate post‐procedural imaging, was achieved in 24/26 cases (92%). Of the two unsuccessful cases, one involved a very large tumour in which incomplete ablation was anticipated; and the other was a caudate lobe lesion where pronounced aortic pulsation prevented adequate tumour immobilisation. All procedures were otherwise uneventful, with no major airway complications, emergency conversion to TLV, reintubation or pneumothorax.

All patients were transferred to the post‐anaesthesia care unit (PACU) for monitored recovery and subsequently to a general surgical ward. All patients were discharged home on postoperative day 1 or 2 following satisfactory clinical and biochemical assessment. No patients required unplanned high‐dependence or intensive care unit admission. Postoperative pain was generally mild, manageable with oral paracetamol and non‐steroidal analgesics. No patient required patient‐controlled analgesia or regional anaesthetic techniques.

## Discussion

This retrospective service evaluation demonstrates that DLT‐OLV is a practical, safe and effective airway strategy for hepatic histotripsy. With a success rate of 81% for DLT placement and 92% of patients ultimately managed with OLV via DLT or bronchial blocker, this approach provided reliable respiratory motion control. Our DLT insertion success rate with the conventional 90 degree counter clockwise rotation is comparable to the figures reported in the literature [15, 16]. Technical tumour ablation success was high at 92%, and all desaturation episodes were manageable with standard interventions without conversion to TLV. To our knowledge, this represents the first published clinical data on OLV for hepatic histotripsy.

The existing literature on anaesthetic management for hepatic histotripsy has focused on HFJV or non‐specified motion‐controlled techniques [1, 2, 8]. High‐frequency jet ventilation offers near‐apnoeic conditions, but it requires dedicated jet ventilators, trained personnel and carries risks of hypercapnia, haemodynamic instability, barotrauma and gas trapping [7, 8, 9, 10]. The resource implications of establishing and maintaining an HFJV service may be substantial for centres performing relatively low volumes of histotripsy.

Double‐lumen tube one‐lung ventilation uses equipment and skills that are widely available in thoracic anaesthesia but carries recognised risks, including hypoxaemia, tube malposition and airway trauma, in addition to postoperative sore throat and hoarseness [17, 18]. In our small retrospective series, DLT‐OLV allowed dynamic adjustment of lung collapse and re‐inflation to optimise ultrasound windows for histotripsy and transient hypoxaemia was managed with standard rescue strategies, suggesting that OLV for histotripsy can be integrated into existing thoracic anaesthetic practice in appropriately selected patients. High‐frequency jet ventilation remains an important alternative motion‐management technique and has been shown to reduce respiratory motion and improve histotripsy precision in experimental and interventional settings; our findings therefore support DLT‐OLV as one feasible option rather than a superior replacement for HFJV.

The retrospective, single‐centre design and absence of a comparator group limit generalisability and preclude direct efficacy comparisons. The sample of 26 patients is modest; however, this cohort reflects the complete initial institutional experience during the first 7 months of programme establishment and is appropriate for a service evaluation describing a novel clinical pathway. Liver motion and diaphragmatic excursion were not measured directly; instead, motion control was inferred indirectly from technical ablation success, acknowledging preclinical evidence that respiratory motion can distort histotripsy treatment zones and reduce treatment precision [8, 19].

Double‐lumen tubes are larger and more difficult to insert than single‐lumen tubes and have been associated with higher rates of postoperative sore throat and airway injury compared with standard tracheal tubes [20]; insertion failed in five cases (19%), predominantly due to anatomical reasons, highlighting the importance of careful pre‐operative airway assessment and the need for a planned alternative airway strategy. In patients with predicted difficult DLT insertion upon conventional airway assessment by an anaesthetist, it may be preferable to secure the airway with a single‐lumen tube and then place a bronchial blocker under bronchoscopy guidance, as recommended in thoracic anaesthesia practice [21, 22, 23, 24].

All patients in our cohort were admitted overnight after histotripsy, though a case report suggested selected patients might be able to be discharged on the same day; our practice might reflect a cautious local approach during the initial implementation phase. However, we did not attempt to evaluate suitability for same‐day discharge in this service evaluation.

Prospective, multicentre randomised controlled trials comparing DLT‐OLV and HFJV across tumour locations and sizes are warranted incorporating objective liver motion assessment, patient‐reported outcomes and health economics analyses of equipment and training costs. Identification of successful DLT placement and desaturation risk would refine patient selection and support individualised airway planning.

## Conclusion

Double‐lumen tube one‐lung ventilation was used to support hepatic histotripsy in this retrospective single‐centre series and we describe its practical application, observed oxygenation profile and immediate technical results in routine practice. Within the limitations of a small, non‐comparative service evaluation, these findings suggest that DLT‐OLV is a feasible airway and motion‐control strategy for liver histotripsy in centres with the necessary anaesthesia expertise, but they do not establish superiority over HFJV or other approaches and should be interpreted as hypothesis‐generating.
